# Empathy and Coping: Older Adults’ Social Encounters and Mood Throughout the Day

**DOI:** 10.1093/geroni/igaa057.2187

**Published:** 2020-12-16

**Authors:** Meng Huo, Yee To Ng, Kira Birditt, Karen Fingerman

**Affiliations:** 1 University of California, Davis, Davis, California, United States; 2 The University of Texas at Austin, Austin, Texas, United States; 3 University of Michigan, Ann Arbor, Michigan, United States

## Abstract

Scholars have proposed empathy as a key characteristic of strong social ties, but little is known about the role empathy plays when tensions occur in these ties. We examined whether older adults’ empathy was associated with their (a) coping strategies for interpersonal tensions, and (b) momentary mood when tensions occurred throughout the day. Data were from the Daily Experiences and Well-being Study. Older adults (n = 302) rated empathy, reported avoidant, constructive, and destructive coping strategies, and indicated tensions and mood every 3 hours each day over 5 to 6 days. More empathic older adults used constructive strategies more often and destructive strategies less often than less empathic older adults. Interpersonal tensions were associated with reduced positive mood throughout the day, but this link was attenuated by older adults’ empathy. This study advances our understanding of empathy and social experiences in later life with a focus on the negative moments.

